# Environmental Factors Influencing Gene Transfer Agent (GTA) Mediated Transduction in the Subtropical Ocean

**DOI:** 10.1371/journal.pone.0043506

**Published:** 2012-08-15

**Authors:** Lauren D. McDaniel, Elizabeth C. Young, Kimberly B. Ritchie, John H. Paul

**Affiliations:** 1 College of Marine Science, University of South Florida, St. Petersburg, Florida, United States of America; 2 Mote Marine Laboratory, Sarasota, Florida, United States of America; King Abdullah University of Science and Technology, Saudi Arabia

## Abstract

Microbial genomic sequence analyses have indicated widespread horizontal gene transfer (HGT). However, an adequate mechanism accounting for the ubiquity of HGT has been lacking. Recently, high frequencies of interspecific gene transfer have been documented, catalyzed by Gene Transfer Agents (GTAs) of marine α-Proteobacteria. It has been proposed that the presence of bacterial genes in highly purified viral metagenomes may be due to GTAs. However, factors influencing GTA-mediated gene transfer in the environment have not yet been determined. Several genomically sequenced strains containing complete GTA sequences similar to *Rhodobacter capsulatus* (RcGTA, type strain) were screened to ascertain if they produced putative GTAs, and at what abundance. Five of nine marine strains screened to date spontaneously produced virus-like particles (VLP's) in stationary phase. Three of these strains have demonstrated gene transfer activity, two of which were documented by this lab. These two strains *Roseovarius nubinhibens* ISM and *Nitratireductor* 44B9s, were utilized to produce GTAs designated RnGTA and NrGTA and gene transfer activity was verified in culture. Cell-free preparations of purified RnGTA and NrGTA particles from marked donor strains were incubated with natural microbial assemblages to determine the level of GTA-mediated gene transfer. In conjunction, several ambient environmental parameters were measured including lysogeny indicated by prophage induction. GTA production in culture systems indicated that approximately half of the strains produced GTA-like particles and maximal GTA counts ranged from 10–30% of host abundance. Modeling of GTA-mediated gene transfer frequencies in natural samples, along with other measured environmental variables, indicated a strong relationship between GTA mediated gene transfer and the combined factors of salinity, multiplicity of infection (MOI) and ambient bacterial abundance. These results indicate that GTA-mediated HGT in the marine environment with the strains examined is favored during times of elevated bacterial and GTA abundance as well as in areas of higher salinity.

## Introduction

The ability to adapt to changing conditions and to evolve is a vital property of all forms of life. Although microbes lack sophisticated sexual reproduction systems, they still manage to exchange genetic material with other bacteria on a regular basis, as evidenced by the genetic signatures of horizontal gene transfer [1], [2], [3], [4]. The three currently described mechanisms of horizontal microbial gene transfer are transformation, conjugation and transduction. However, another mechanism mediated by the virus-like Gene Transfer Agents (GTAs) has recently been demonstrated to be active in natural environments and to have the potential to catalyze very high levels of gene transfer [5]. Nonetheless, the proportion of GTAs in natural viral assemblages and the factors influencing GTA-mediated transduction in the environment remain uninvestigated.

GTAs were initially described in the α-Proteobacterial strain *Rhodobacter capsulatus* (formerly *Rhodopseudomonas capsulata*) [6]. In the type strain *R. capsulatus* GTAs are designated RcGTA and resemble small, tailed bacteriophage-like particles. However, rather than encapsidating their own viral genome, they package random approximately 4 kb pieces of the host DNA [7]. Therefore, the RcGTA is a genetic exchange vector under the control of host proteins within a two-component signal transduction system [8], [9]. In contrast to lytic bacteriophages, spontaneous production of phage-like GTA particles in stationary phase growth without detectable host lysis is a hallmark of GTAs [7], [10]. Although the exact mechanism of GTA release has not been determined to date [9], one possible explanation is that GTAs may be released due to lysis of a small subset of the total population. Several similar GTA-like systems have been discovered in diverse prokaryotic laboratory strains including *Brachyspira hyodyseneriae* with the GTA designated VSH-1 [11], *Desulfovibrio desulfuricans* with Dd1 [12], *Bartonella* species producing BLP [10], [13] and the Archeaon *Methanococcus voltae,* which produces VTA [14], [15]. These particles range greatly in size, morphology and the amount of host DNA they package [10].

Most investigation of GTAs has been from systems in culture. Nevertheless, data from viral metagenomes may provide clues to the prevalence of GTAs in natural viral assemblages. It has often been observed that environmental viral metagenomic databases frequently contain many bacterial genes and it has generally been presumed that this was due to contamination by bacterial DNA. However, it has recently been proposed that this may be due, at least in part, to the presence of GTAs [16]. What the proportion of the viral population that is composed of GTAs has not been determined.

Genome sequencing projects have uncovered many similar GTA-like gene clusters in diverse α-Proteobacteria, which appear to be highly conserved and vertically inherited within this lineage [10], . The degree of conservation of viral-like GTA genes is so great that they have been used as diagnostic markers for the order *Rhodobacterales* in the environment [18], [19]. Recent evidence from sequencing of a lytic phage of *Roseobacter denitrificans* provides strong support for the contention that GTAs are relic prophages [20]. Besides the type strain *R. capsulatus*, production of GTAs with intraspecific gene transfer has been documented in the lab setting using the strain *Ruegeria pomeryoi* DSS-3 (formerly *Silicibacter pomeroyi*) [21] and expression of the *R. capsulatus*-like GTA major capsid protein gene has been detected in the RNA transcripts of several cultured strains of *Rhodobacterales* [18].

Production of functional GTAs with interspecific gene transfer capabilities, utilizing two different α-Proteobacterial species, *Roseovarius nubinhibens* ISM and *Nitratireductor* strain 44B9s, has recently been documented [5]. We undertook this work to investigate two main questions. Firstly, to assess the level of GTA production in cultured strains to attempt to determine how many cultured α-Proteobacterial strains produce GTAs and to constrain a first estimate of the number of GTAs in natural viral assemblages. Secondly, to determine if any easily measurable environmental factors appear to facilitate the process of GTA-mediated gene transfer in the environment. Gene transfer experiments were performed over a wide range of times and conditions. Additionally, because many GTA-containing strains also contain prophages [17], [22] prophage induction experiments were conducted concurrently to determine if similar environmental conditions are favorable for both GTA-mediated gene transfer and lysogeny in natural populations.

## Results

**Figure 1:**
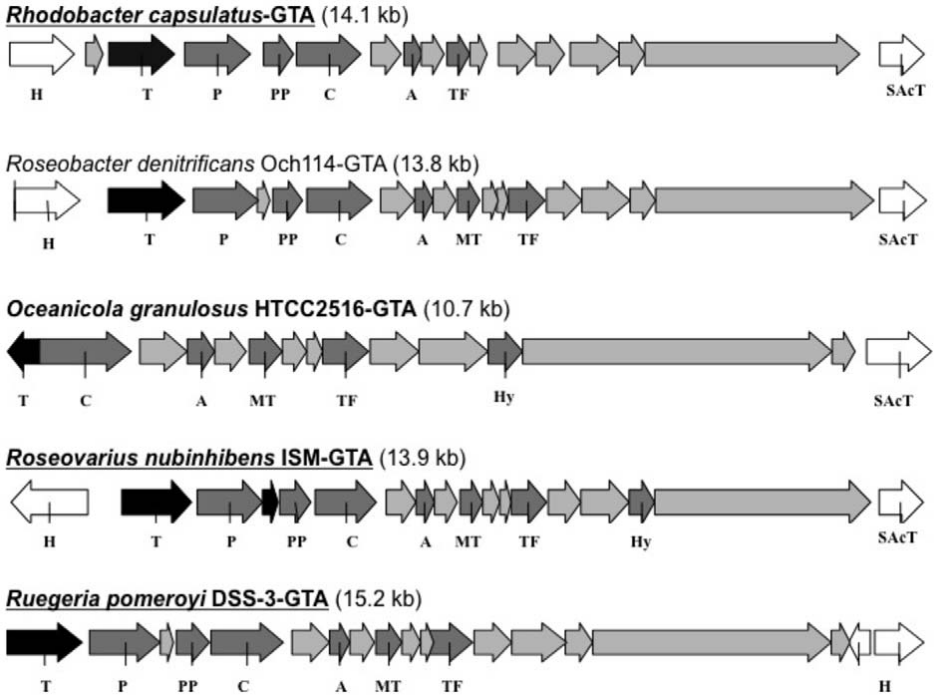
GTA Gene Maps: GTA gene maps from representative, fully sequenced strains used in this study. *Rhodobacter capsulatus* is the type strain. Strain name in bold indicates a strain that produces putative GTA particles in stationary phase growth. Strain name underlined indicates gene transfer activity of the particles has been demonstrated experimentally. The size of the GTA coding region (kilobase pairs) is indicated in parentheses next to the strain name. The GTA terminase is color-coded black, dark gray indicates GTA genes with identified putative functions, light gray indicates open reading frames with sequence conservation but unknown functions and white indicates host-associated genes (H  =  Host Associated, T  =  GTA terminase, C  =  GTA major capsid, P  =  GTA portal protein, PP  =  GTA pro-head protease, A  =  head-tail adaptor, MT  =  Major tail protein, TF  =  Tail fiber, Hy  =  cell wall hydrolase, SAcT  =  host Serine O-Acetyl transferase).

Many sequenced marine α-Proteobacterial isolates have been observed to contain identifiable GTA-like gene clusters [17], (Figure 1). Several of these sequenced, GTA containing α-Proteobacterial strains were acquired for this study (Table 1). To date, four marine strains have been documented to spontaneously produce GTA-like particles with no precipitous decline in host cell abundance after entering stationary phase growth [5], [21]. For this study seven marine α-Proteobacterial strains were screened for the spontaneous production of virus-like particles during stationary phase (Table 1). Of these seven strains *Oceanicola granulosus*, *Roseovarius nubinhibens* ISM, *Ruegeria mobilis* strain 45A6 and *Nitratireductor* strain 44B9s were observed to produce particles in this way and the particles were considered potential GTAs. The day of maximal production of GTAs was different for each strain, but reproducible within that strain [23], [24]. Maximum levels of GTAs produced for the strains examined are listed in Table 1. For the determination of GTA production as a function of cell abundance experiments were performed with paired GTA and cell counts. At the day of maximal GTA production for the *R. nubinhibens* and the *R. mobilis* 45A6 strains, GTA particles and cells were enumerated. In these experiments the GTA counts were slightly lower than the observed maxima. For the *R. nubinhibens* ISM strain the GTA particles were measured at 3.5×10^8^ ml^−1^ and the cell counts were 2.8×10^9^ ml^−1^ with GTA particles equating to 10.9% of cell abundance. For the *R. mobilis* 45A6 strain the GTA abundance was 5.1×10^8^ ml^−1^ and the cell counts were 1.4×10^9^ ml^−1^ with GTA particles equating to 30% of the cell abundance.

**Table 1:** Sequenced Alpha-Proteobacterial strains containing GTA gene cassettes and screened for GTA production over time.

Isolate Name	ATCC strain number	Maximum GTA production	Genome Accession Number (Refseq)	Locus tag- GTA Terminase/GTA ORF 15	Growth Media	Preferred temp
*Rhodobacter capsulatus* SB1003WT	Gift from Oregon State University	yes (type strain, 10^5^ gene transfer units ml^−1^), Solioz et.al., 1975	NC_ 014034	RCAP_rcc01683 RCAP_rcc01698	PYE, YPS, LB or OM	30°C aerobic, or anaerobic with light
***Roseobacter denitrificans*** ** OCH114**	ATCC® 33942^TM^	not observed	NC_ 008209	RD1_3034 RD1_3016	Marine Broth 2216	26°C aerobic
***Oceanicola granulosus*** ** HTCC2516**	ATCC® BAA861^TM^	yes, (2.9×10^6^ ml^−1^)	NZ_ AAOT00000000	OG2516_04279 OG2516_04219	Marine Broth 2216	30°C aerobic
***Roseovarius nubinhibens*** ** ISM**	ATCC® BAA591^TM^	yes, (1×10^9^ ml^−1^)	NZ_ AALY00000000	ISM_05135 ISM_05210	Marine Broth 2216	30°C aerobic
*Ruegeria* (*Silicibacter*) *pomeroyi* DSS-3	ATCC® 700808^TM^	yes, (×10^8^ ml^−1^) Biers et.al., 2008	NC_ 003911	SPO2266 SPO2250	Marine Broth YTSS	28°C aerobic
***Oceanicaulis alexandrii***	Gift from Oregon State University	not observed	NZ_ AAMQ00000000	OA2633_14805 OA2633_14855	Marine Broth 2216	20°C aerobic
***Aurantimonas manganoxydans*** ** SI85-9A-1**	ATCC® BAA-1229	not observed	NZ_ AAPJ00000000	SI859A1_01033 SI859A1_08129	#2584 Broth: buffered marine media with Mn	30°C aerobic
*Parvularcula bermudensis*	ATCC® BAA594^TM^	pending	NC_ 014414	PB2503_08059 PB2503_08129	Marine Broth 2216	30°C aerobic
***Nitratireductor*** **, strain 44B9s**	Isolated from *Symbiodinium* associated with a *Gorgonian*	yes (2×10^9^ ml^−1^)	N/A	N/A	Marine Broth 2216	26°C aerobic
***Ruegeria mobilis, *** **strain 45A6**	Isolated from a Clade D2 *Symbiodinium* associated with a Foraminifera	yes (1×10^9^ ml^−1^)	Pending	Pending	Marine Broth 2216	26°C aerobic

The strains screened for this study are indicated in bold type. Maximum abundance of GTA particles observed in parentheses.

The strains *Roseovarius nubinhibens* ISM, and *Nitratireductor* strain 44B9s were mutagenized with Tn5 [25], which has a known sequence and encodes genes for both Kanamycin and Streptomycin resistance. Since GTAs function by packaging random pieces of host DNA the strains mutagenized with Tn5 would produce GTA particles with some proportion containing Tn5 sequences including the genes for antibiotic resistance. These mutagenized strains were subsequently used as donor strains in production of GTAs for environmental gene transfer experiments.

**Figure 2:**
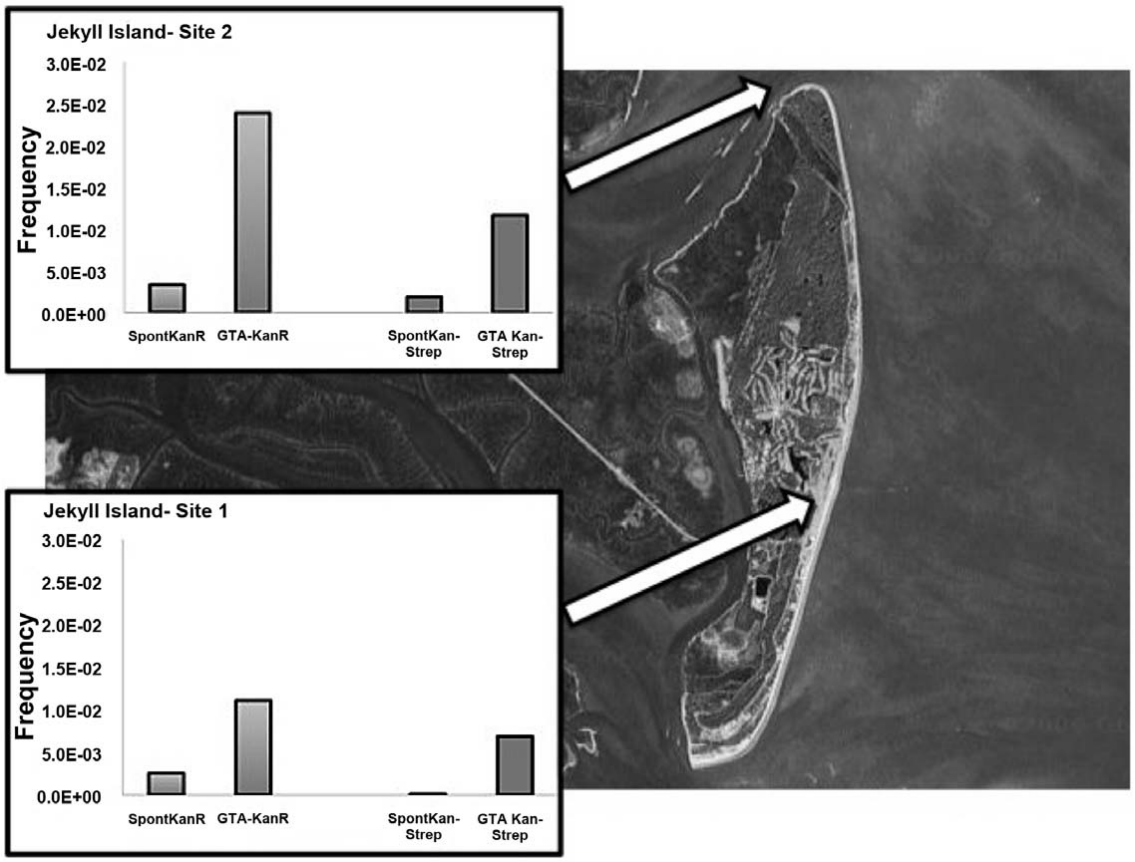
RnGTA-mediated gene transfer experiments at two sites off Jekyll Island, GA: The background is a satellite map of the island with the arrows indicating the sampling sites. The insets show the single antibiotic gene transfer frequency on the left side of the panel in gray and the double antibiotic gene transfer frequency on the right side of the panel in dark gray. (SpontKanR  =  spontaneous kanamycin revertants; SpontKan-Strep  =  spontaneous kanamycin and streptomycin revertants; GTA-KanR  =  RnGTA GTA treated kanamycin resistance; GTA Kan-Strep  =  RnGTA treated kanamycin and streptomycin resistance).

Sampling of natural microbial assemblages was undertaken in several differing natural environments and at varying times of year to determine if GTA-mediated gene transfer could be observed in microbial populations from diverse environments. Samples from a coastal environment in Georgia were obtained in October (Figure 2). For Site 1 the ambient viral abundance was 2.2×10^7^ viruses ml^−1^ and the bacterial abundance was 1.2×10^6^ cells ml^−1^, yielding a VBR of 18.3. The calculated GTA MOI was 332 GTAs cell^−1^ At Site 2 the measured viral and bacterial abundances were similar at 2.05×10^7^ viruses ml^−1^ and 1.6×10^6^ cells ml^−1^, respectively. For this site the VBR was 12.8 and the calculated GTA MOI was 254 GTAs cell^−1^. Interestingly, both of these sites had a high percentage of cultivable bacteria with a calculated 60% of the bacterial abundance at Site 1 forming colonies and 25% at Site 2, however this calculation was based on plate counts after the experimental overnight incubation and some growth of the microbes in the samples is likely. At both of these sites a statistically significant increase in both single (Kanamycin) and double (Kanamycin and Streptomycin) antibiotic resistance was observed in the GTA treated samples in comparison to controls. Total viable counts were statistically indistinguishable between treatments and controls (Figure 2).

All of the spontaneous double antibiotic resistant colonies from the controls were screened by PCR for the presence of the Tn5 Streptomycin kinase gene as well as several double antibiotic resistant isolates from the replicate GTA treated samples. The gene was not recovered from any of the spontaneous revertants but the exact match to the Tn5 gene was recovered from 11% of the screened treatment colonies. The high rate of double antibiotic resistance is extremely improbable by chance; nonetheless, only 11% of the viable colonies produced the expected Streptomycin kinase gene. It is possible that even though the active sites of the genes were successfully transferred, some modifications, truncations, or re-arrangements may have occurred. This type of gene alteration has been documented in the past for genes transferred to marine bacterial populations by natural transformation [26].

Because of the common association between the coral endosymbiont *Symbiodinium* and α-Proteobacteria [27], GTA-mediated gene transfer was examined in a reef environment. The samples with their associated microbes from the vicinity of the Looe Key reef were acquired in September. Sampling was performed immediately adjacent to the reef surface. In this sample the ambient bacterial abundance was 1.16×10^6^ ml^−1^ and the viral abundance was 2.34×10^7^ ml^−1^ measured by flow cytometry, yielding a virus to bacteria ratio of 20.2. The calculated GTA MOI was 150 GTAs cell^−1^.

**Figure 3:**
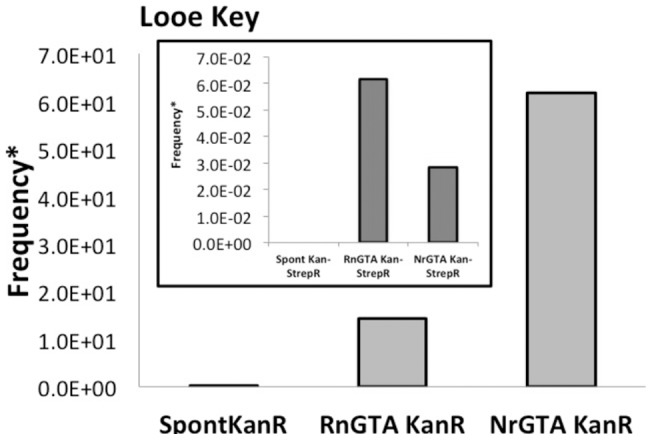
GTA-mediated gene transfer in the reef environment: RnGTA and NrGTA mediated gene transfer frequencies at a site near Looe Key Reef. Main figure indicates single antibiotic frequencies and inset indicates double antibiotic frequencies. The asterisk indicates that the viable/culturable counts for that experiment were below the detection limit (200 cfu ml^−1^) so the frequency was calculated using the calculated detection limit. Note that the frequency calculated in this manner is an underestimate of the actual frequency. Also note, there were no spontaneous double antibiotic revertants at this site.

The water samples were incubated with GTAs produced by both the *R. nubinhibens*: Tn5 (RnGTA) and the *Nitratireductor* 44B9s: Tn5 (NrGTA) donor strains. A statistically significant increase in antibiotic resistance was observed in the treated samples for both GTAs and for both single and double antibiotic selection (Figure 3). In this experiment parallel samples tested for prophage induction also contained inducible lysogens in the ambient microbial population as evidenced by a statistically significant increase in virus abundance in the Mitomycin C treated samples (Table 2). The isolates from these experiments were not screened for Tn5 sequences.

**Table 2:** Environmental parameters measured in conjunction with cruise GTA-mediated gene transfer and prophage induction experiments.

Station	4	5	LK	2	7
Environment Type	Oligotrophic	Upwelling	Coral Reef	Near Shore	River Mouth
Location	27.42.585N 83.27.842W	27.41.694N 84.05.402W	24.33.138N 81.23.028W	27.45.460N 82.36.570W	27.51.464N 82.27.368W
Temperature (°C)	16	16	28	16	20
Salinity	39	39	38	34	27
Ambient viruses (ml^−1^)	3.7×10^7^	2.4×10^7^	2.34×10^7^	1.04×10^8^	1.5×10^8^
Prophage Induction (+/−)	No	No	Yes	No	No
Ambient bacteria (ml^−1^)	1.3×10^6^	1.2×10^6^	1.16×10^6^	2.5×10^6^	5.01×10^6^
Percent Culturable	0.02%	0.02%	>1%	1.60%	3.40%
Virus to bacteria ratio (VBR)	27.9	19.3	20.1	41.6	29.9

To investigate further, a short research cruise was undertaken with sampling of a series of four stations in the vicinity of the Gulf of Mexico. The stations were selected to represent a gradient of conditions over a short temporal scale including oligotrophic, oligotrophic with upwelling, near shore and estuarine river mouth stations (Table 2). In this case, the samples were obtained during late winter (February). The gradient in conditions can be observed in the decreasing salinity and increasing viral and bacterial abundances moving from the oligotrophic areas toward the more eutrophic river mouth inside Tampa Bay (Table 2).

**Figure 4:**
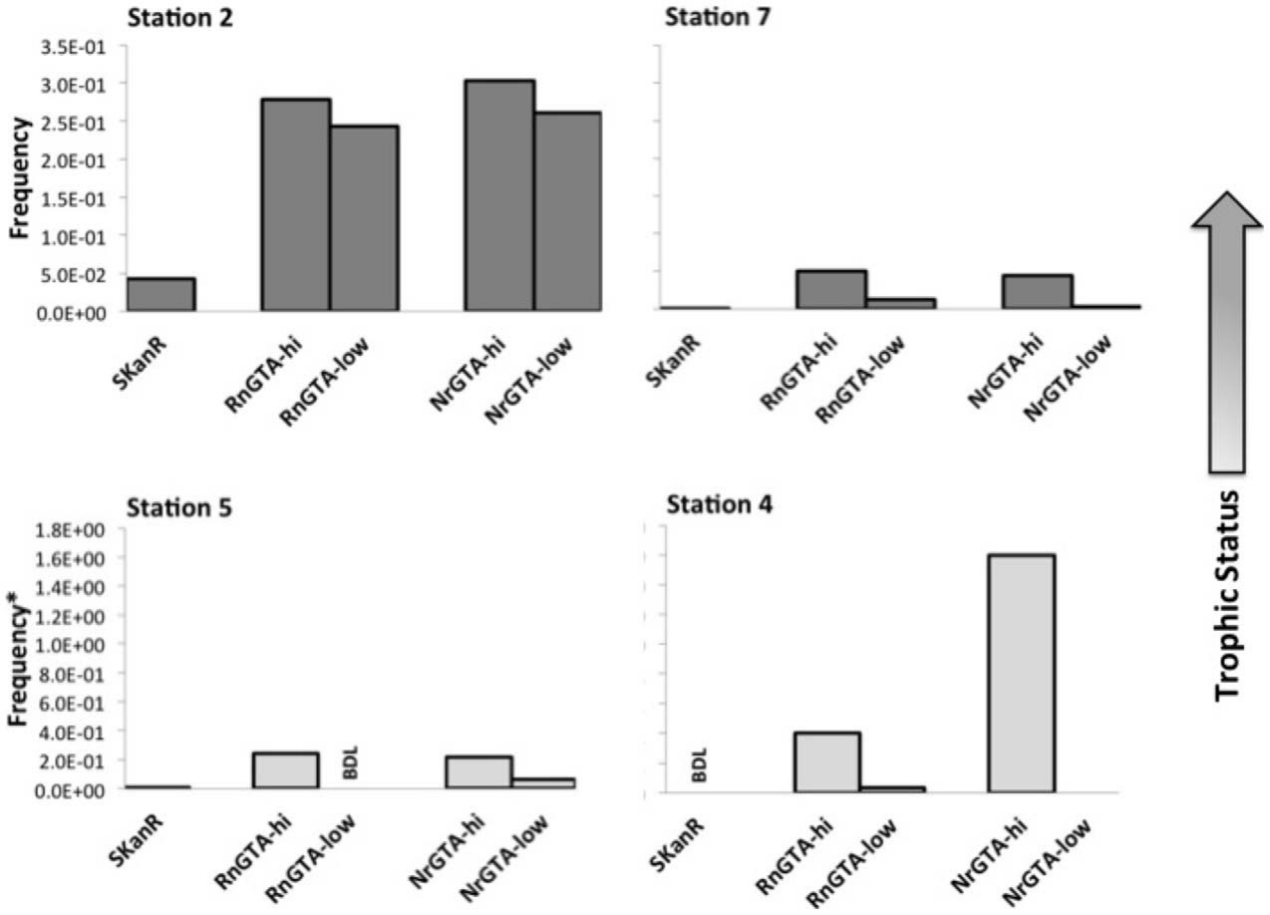
GTA-mediated gene transfer in the Gulf of Mexico: RnGTA and NrGTA low and high multiplicity of infection (MOI) experiments from the Gulf of Mexico/Tampa Bay cruise. The asterisk indicates that the viable/culturable counts for that experiment were below the detection limit (200 cfu ml^−1^) so the frequency was calculated using the calculated detection limit. Note that the frequency calculated in this manner is an underestimate of the actual frequency. Stations are as indicated in Table 2 (SKanR  =  spontaneous kanamycin revertants; RnGTA-low  =  RnGTA treated, low MOI; RnGTA-hi  =  RnGTA treated, high MOI; NrGTA-low  =  NrGTA treated, low MOI; NrGTA-hi  =  NrGTA treated, high MOI).

Similarly to the reef experiment, samples were incubated with GTA particles produced using both donor strains. In addition, both high and low MOI treatments for each GTA type were included. At the oligotrophic station 4, no spontaneous antibiotic revertants were cultured and the GTA treated samples did produce some colonies demonstrating resistance to Kanamycin (Figure 4, Station 4). However, statistical significance of the result could not be confirmed due to high variability in the data. At the oligotrophic station with upwelling (Figure 4, Station 5), GTA-mediated transfer was observed with the RnGTAs at a high MOI and with the NrGTAs at both the low and high MOIs. At the two near-shore stations (Stations 2 and 7), gene transfer was even more prevalent, primarily due to easier detection because of the higher numbers of cultivable bacteria. Note the higher ratios of cultivable bacteria in comparison to total bacterial counts at stations 2 and 7 as compared to the oligotrophic stations (Table 2). Higher levels of gene transfer were also observed with the high MOI treatments in comparison to the similar low MOI treatments at all of the stations sampled. Several isolates (a total of 67) from the spontaneous antibiotic resistant controls and the two GTA treatments were screened by PCR for the Tn5 Streptomycin kinase gene. No amplicons were recovered from the spontaneous revertants. However, the correct amplicon was recovered from 12% of the RnGTA treated isolates and 17.6% of the NrGTA treated isolates.

In the all of the February cruise samples parallel prophage induction experiments were performed. In these samples both the bacterial and viral abundances were measured in the control and Mitomycin C treatments. Although a decrease in bacterial abundances was observed at stations 4, 2 and 7 in response to the Mitomycin C treatment, in no case was a corresponding statistically significant increase in viral abundance observed. Thus, prophage induction did not occur at any of the sampled stations (Table 2).

Statistical comparison of the GTA-mediated gene transfer frequency and measured environmental parameters for fourteen separate experiments in which GTA-mediated gene transfer was detected, including those discussed in detail above. The measured salinity ranged from 27 to 39, the temperature ranged from 16–30.5°C and the virus to bacteria ratio ranged from 13–51. The average ambient bacterial abundance from all experiments was 1.6×10^6^ cells ml^−1^ (range 3.3×10^5^–5.01×106 ml^−1^), and the average ambient viral abundance was 4.4×10^7^ ml^−1^ (range 8.3×10^6^–1.04×10^8^ ml^−1^). Multiple correlation analysis indicated one statistically significant correlation with the GTA-mediated gene transfer frequency, which was an inverse relationship between gene transfer and temperature (r = −0.661, P = 0.037, n = 14). This result suggested that more gene transfer occurred in cooler conditions. Modeling of the GTA-mediated gene transfer frequency by stepwise regression indicated that the environmental parameters most explanatory of increasing gene transfer frequency were the combined parameters of higher salinity, increasing GTA dose (MOI) and lower ambient bacterial abundance with a combined P value  = 0.019 and an adjusted R^2^ of 0.992 for this model. Although temperature was correlated with gene transfer frequency, it was not a strong explanatory factor in the best-fit model.

## Discussion

Our examination of GTA gene clusters from sequenced α-Proteobacteria revealed a high degree of synteny, even among distantly related strains (Figure 1). Based on amino acid alignments of GTA genes from sequenced strains, two of the most conserved features are the GTA terminase large subunit and the capsid protein (data not shown). Another distinctive feature is the large open reading frame (ORF) annotated as ORF 15 in the type strain *R. capsulatus*, but in some cases is annotated as “host-specificity factor” (Figure 1), perhaps homologous to phage tail fiber proteins [20]. As far as we are aware, the exact function of this large ORF has not been conclusively determined, although recent sequencing of a lytic roseophage suggests it may encode a tail fiber protein [20].

Our comparison of nucleotide level alignments of each of the GTA genes from sequenced strains indicates the highest level of sequence conservation is found within the GTA terminase (data not shown). Interestingly, many of these large subunit terminase genes do not have an identifiable associated small-subunit terminase. In lytic viruses the terminase enzyme typically has two subunits; the large subunit, which cuts and packages the DNA, and the small subunit, which has sequence specificity and determines where to cut. It has been hypothesized that the lack or loss of the small subunit, which determines DNA sequence specificity, may be key to the packaging of random pieces of host DNA, which is characteristic of GTAs [17]. Another notable feature of many of the GTAs is their common proximity to the host gene for a serine O acetyl-transferase (Figure 1), an indication of vertical inheritance of GTAs. One difference between GTAs is that some contain an identifiable cell wall hydrolase and some do not.

These experiments demonstrated that functional GTAs are produced by some, but not all, strains with a bio-informatically identified GTA gene cassette. The fact that the other strains were not observed to produce GTA particles does not mean that they would not do so under different environmental conditions. It is highly likely that the conditions under which they produce GTAs were not adequately replicated in the laboratory setting.

In addition to containing GTAs, many of these strains also carry identifiable prophages. In the cultured isolates investigated so far, prophage induction appears to occur under differential conditions to that for GTA production [5], [17], [22]. Because of this, we investigated whether there was a relationship between lysogeny, or any other measured environmental parameter, and GTA-mediated gene transfer in natural microbial assemblages from a variety of environments.

The first concurrent GTA and prophage induction experiments in the reef environment demonstrated both lysogeny, evidenced by Mitomycin C stimulated prophage induction as well as GTA-mediated gene transfer (Table 2). These first experiments suggested similar conditions may favor lysogeny and GTA-mediated gene transfer.

In another set of experiments performed during the winter, over a wide range of trophic conditions it was observed that higher levels of gene transfer were measured when higher dosages of GTAs were used in all similar experiments. This is consistent with previously observed linear response of increasing gene transfer to increasing GTA dose in culture experiments [5].

In contrast, in these samples, no prophage induction was detected (Table 2). This was somewhat surprising, since previous seasonal studies have indicated that lysogeny is commonly, but not always, observed in similar environments during the winter season [28]. For these paired experiments we observed that GTA-mediated gene transfer and prophage induction were not observed together in the same samples. From this observation we concluded that the environmental factors favoring GTA-mediated gene transfer differed somewhat from those favoring lysogeny in the natural microbial assemblages present for those samples. This is consistent with the observation in cultured strains that the two processes, while not necessarily being mutually exclusive, are favored by varying conditions, which in the natural environment may include physiological differences or shifts in the microbial community composition.

Interestingly, correlation analysis of all of the environmental experiments and the measured environmental parameters revealed a statistically significant inverse relationship between increasing GTA-mediated gene transfer frequency and temperature. Although temperature was the only significantly correlated variable by simple multiple correlation analysis, modeling of the level of environmental GTA-mediated gene transfer, expressed as the frequency and all measured environmental parameters together indicated the combined factors of salinity, MOI and ambient bacterial abundance best explained the GTA-mediated gene transfer frequencies. This suggests that increasing GTA dose, expressed as MOI is related to increasing levels of gene transfer. This is also consistent with experiments in culture that demonstrate a linear relationship between increasing MOI and gene transfer frequency [5].

We have only performed a few studies in low salinity environments, and the mechanism for lower levels of GTA-mediated transfer under these conditions has yet to be determined. However, the ambient bacterial abundance was negatively correlated with salinity (r = −0.944, P<0.001) and the relationship between GTA-mediated gene transfer and salinity may be coupled to the differences in the abundance or composition of the associated bacterial community. Alternatively, this result may be due to the fact that the GTAs in use for the experiments to date were produced and isolated from marine strains and may not be adapted for less saline environments such as rivers or lakes. Since α-Proteobacteria are ubiquitous in many environments, it would be informative to investigate this process in a wider range of settings, using strains adapted to local conditions.

If we postulate that GTAs in the environment are produced at a similar abundance as in culture, by about half the GTA-containing strains (see above, Table 1), using the average bacterial abundance of 1.6×10^6^ ml^−1^ and an assumed α-Proteobacteria-related population of 30%, using the lower factor for GTA production of 10% of host abundance would translate to native abundance of approximately 2.4×10^4^ GTA particles ml^−1^. Continuing this extrapolation using the same value for bacteria would lead to an approximated MOI of 0.015. Although increasing GTA dose generally appears to lead to higher frequencies of gene transfer, we have observed GTA-mediated gene transfer at even lower MOIs than 0.01, indicating that GTA-mediated gene transfer could be supported in natural settings.

If we continue our extrapolation using the average ambient viral abundance of 4.4×10^7^ viruses ml^−1^, the estimated GTA abundance (as above) could represent up to an estimated 0.05% of the ambient viral population. This number is highly likely to be an overestimate since it also assumes that half of the *Roseobacter* related bacteria are producing GTAs in a given environment, which is unknown at this juncture. Further research is in progress to attempt to constrain this number experimentally.

This study has demonstrated that GTA-containing strains of cultured *Rhodobacterales* can produce GTA-like particles and participate in cell-free, GTA-mediated gene transfer in both laboratory and environmental settings. Environmental conditions do affect the frequency of GTA-mediated gene transfer with higher frequencies observed at lower temperatures. Statistical modeling of the GTA-mediated gene transfer frequency and all the measured environmental parameters suggested that increasing gene transfer frequency in the marine environment was associated with the combined conditions of higher salinity, higher GTA dose (MOI) and lower ambient bacterial abundance. Prophage induction and GTA-mediated gene transfer were observed in the same sample in one experiment. However, significant gene transfer was observed without presence of prophage induction in three experiments. This indicates that the processes of GTA-mediated gene transfer and lysogeny, while not mutually exclusive, are not necessarily favored by similar environmental conditions.

## Materials and Methods

### Screening of Isolates

Several fully sequenced marine strains of α-Proteobacteria were identified that contained Gene Transfer Agent (GTA) gene cassettes similar to that of the type strain *Rhodobacter capsulatus* (Figure 1), as documented in Paul 2008 [17]. Several of these strains were selected based on the similarity of their GTA gene cluster to the type strain, *R. capsulatus* (Figure 1) and were subsequently obtained from culture collections (Table 1). Each strain was grown to stationary phase in the recommended media at the optimal temperature for that strain. They were monitored for the spontaneous production of viral particles after attaining stationary phase, which is a hallmark of GTA production, by SYBR Gold staining according to established protocols [29]. Host cell density was monitored by absorbance at 600 nm (A600).

Three strains were identified and have been documented to produce functional GTA particles in sufficient quantities to use in gene transfer experiments [5]. Two of the three strains, *Roseovarius nubinhibens* ISM and *Nitratireductor* strain 44B9s, were selected for further experimentation. Both strains were subsequently mutagenized with the transposon Tn5 as previously described [5] in order to confer antibiotic resistance (Kanamycin and Streptomycin) for selection as well as traceable genetic markers [25]. The mutagenized, antibiotic resistant strains *R. nubinhibens*: Tn5 and *Nitratireductor* 44B9s: Tn5 were subsequently used as the donor strains for production of GTA particles in all environmental gene transfer experiments.

### Purification of GTA particles

GTA particles were harvested from each strain at the time of maximal production for the specific strain, coinciding to 5 and 3 days after start of culture growth for *Roseovarius* and *Nitratireductor* respectively. The particles were harvested and concentrated using standard protocols for virus purification [30]. Briefly, the cultures were centrifuged at 9,500× g for 10 minutes at room temperature to remove the cells. The supernatant was subsequently 0.2 μm filtered to remove any remaining host cells. The filtrate was DNase and RNase treated to degrade any free nucleic acids. After that, NaCl was added to a final concentration of 1 M, followed by polyethylene glycol (PEG) to a final concentration of 10% weight to volume. The filtrates containing PEG were refrigerated overnight to aid in precipitation of the GTA particles and then centrifuged at 9,500× g for 20 minutes at 4°C to pellet the particles. The PEG solution was aspirated with a Pasteur pipette and drained completely. The GTA pellets were re-suspended in a small volume of sterile SM buffer [30] then an equal volume of chloroform was added to the samples, thoroughly mixed and then centrifuged in a phase-lock gel tube (5 Prime, www.5prime.com) according to the manufacturer's instructions to remove any traces of PEG. This also served to demonstrate that the particles were not membrane vesicles, as vesicles would have been disrupted by the chloroform treatment. The concentrated GTAs remained in the aqueous fraction, were recovered, and then enumerated by SYBR Gold staining to determine the final concentration and then checked for complete removal of host cells as above. The concentrated particles were also tested for absence of host cells by plating on the same media used for gene transfer experiments (without antibiotics) to rule out the possibility of conjugal transfer. Additionally, they were treated with DNase immediately prior to use in any gene transfer experiment to rule out the possibility of natural transformation occurring.

### Environmental GTA-mediated Gene Transfer

Water column samples containing natural microbial assemblages were utilized as the recipients in GTA-mediated gene transfer experiments. Where prophage induction experiments were performed the same samples were tested concurrently for detectable prophage induction. For each environment tested, the water samples were passed through 50 μm mesh to remove any grazing organisms. In conjunction with gene transfer experiments the environmental parameters measured included: temperature, salinity, ambient bacterial and viral concentrations. In addition, the GTA multiplicity of infection (MOI), ambient virus to bacteria ratios (VBR) and percentage of the microbial population that was culturable were calculated.

For the Jekyll Island experiment, triplicate water samples were obtained from two sites on October 10^th^, 2010. Site 1 was located at 31°3.306′ N ×81°25.139′ W. Site 2 was located at 31°6.958′ N ×81°25.042′ W (Figure 1). For the reef environment water samples were obtained in the vicinity of Looe Key Reef adjacent to the reef surface (Table 2) on September 27^th^, 2009. Surface water samples in a range of conditions were obtained during a research cruise on the R/V Bellows from February 9–11, 2010 (Table 2).

For the GTA-mediated gene transfer experiments, the samples were divided into replicate treatment and controls and then amended with either concentrated GTA particles for the treatment or an equal volume of sterile SM buffer for the controls. GTAs were added at a concentration to achieve an estimated MOI of ten for the High MOI and 0.01 for the low MOI. The actual MOI was calculated based on the ambient bacterial counts and the known concentration of the GTA preps. Ambient viral and bacterial counts were determined from samples preserved with glutaraldehyde, frozen in liquid nitrogen and enumerated upon return to the lab using flow cytometry as previously described [31].

For the GTA experiments, the samples were plated on marine agar supplemented with peptone and yeast extracts [32]. Plates without antibiotics were used to enumerate the number of culturable bacteria expressed as the number of colony-forming units per milliliter of sample (CFU ml^−1^). The samples were also plated on the same nutrient agar containing 1 mg ml^−1^ of Kanamycin for the single antibiotic treatment and 1 mg ml^−1^ of Kanamycin and 1 mg ml^−1^ Streptomycin for the double antibiotic treatment. The number of CFU ml^−1^ detected from the untreated samples determined the level of spontaneous antibiotic resistance. This level was compared to the antibiotic resistant CFU ml^−1^ for the GTA-treated samples. The frequency of antibiotic resistance was calculated by dividing the number of antibiotic resistant CFU ml^−1^ by the total culturable CFU ml^−1^. Gene transfer was considered to have occurred when there was a statistically significant increase in antibiotic resistance in the GTA treated samples in comparison to the controls. A subset of the antibiotic resistant colonies, both GTA treated and spontaneous revertants, were picked and grown in liquid. The isolates that grew were centrifuged and the cellular DNA extracted from the cell pellet using either the ArchivePure DNA extraction kit (5 Prime, www.5prime.com) or the Wizard Genomic DNA kit (Promega, www.promega.com) according to the manufacturer's instructions. Transfer of the Tn5 Streptomycin kinase gene via GTA to a proportion of the treated isolates and its absence in the spontaneous revertants was verified by both PCR and sequencing of the amplicons as previously described [5].

For prophage induction, duplicate samples were split into treatment and control flasks. Treatment flasks were amended with Mitomycin C to a final concentration of 1 μg ml^−1^ and no amendment for the controls. All samples were incubated at ambient temperature in the dark for 24 hours. For determination of lysogeny by prophage induction, triplicate Mitomycin C treatment and control samples were preserved with glutaraldehyde and frozen in liquid nitrogen. Viral and bacterial counts for both the treatment and control samples were determined by flow cytometry as above. Prophage induction was indicated by a statistically significant increase in viruses in the Mitomycin C amended samples in comparison to their paired controls.

All statistical analysis was performed using Minitab v.13 software (www.minitab.com). Significance of environmental gene transfer experiments was determined by comparing CFUs ml^−1^ in treatment and control samples by ANOVA with Tukey's post-hoc test. GTA-mediated gene transfer frequency and all measured environmental parameters for all significant experiments were compared by multiple correlation analysis. Modeling of the factors affecting gene transfer frequency in the environment was performed by stepwise linear regression using an alpha-value of 0.15 to enter or remove a parameter from the best-fit model.
